# Dangerous medicines: Unproven AIDS cures and counterfeit antiretroviral drugs

**DOI:** 10.1186/1744-8603-4-5

**Published:** 2008-02-27

**Authors:** Joseph J Amon

**Affiliations:** 1 HIV/AIDS Program, Human Rights Watch, 350 Fifth Ave., 34^th ^Floor, New York, NY 10118, USA

## Abstract

**Background:**

Increasing access to antiretroviral therapy (ART) is a critical goal endorsed by the United Nations and all of its member states. At the same time, anecdotal accounts suggest that the promotion of unproven AIDS 'cures' and remedies are widespread, and in the case of The Gambia, Iran and South Africa, have been promoted by governments directly. Although a range of legislative and regulatory measures have been adopted by some governments, and technical assistance has been provided by international agencies to address counterfeit medicines generally, the threat of counterfeit antiretroviral drugs is not being addressed.

**Discussion:**

Countries, charged with fulfilling the right to health and committed to expanding access to ART must explicitly recognize their obligation to combat unproven AIDS treatments and ensure the availability of a safe and efficacious drugs supply. International donors must help support and coordinate these efforts.

## Background

In the last few years governments around the world have pledged to massively scale up the delivery of antiretroviral drugs (ARVs) to achieve universal access to all. To date attention has focused on how to finance this effort and how to strengthen health care delivery systems, including how to conduct mass HIV testing programs and increase the number of health care providers worldwide. Less attention has been paid to the responsibilities of governments and international agencies to address the widespread prevalence of unproven AIDS 'cures' and the threat of counterfeit ARVs.

### Marketing AIDS 'cures'

Treatments to cure HIV/AIDS have been falsely promoted since AIDS was first identified and claims of cures continue in spite of the development of ARVs, which can dramatically slow the progression to AIDS but do not rid the body of HIV. The uncertainty and fear engendered by this incurable, stigmatizing and life-threatening disease can make people easy prey to promises of cures. But in addition to fear, there are specific factors, related to government functioning, that facilitate false claims of HIV cures, including: little or no access by people living with HIV and those populations most at risk to health care generally and ARVs specifically; stigmatizing and discriminatory treatment of people living with HIV/AIDS (PLWHA) in health care settings; high prices for legitimate pharmaceuticals when available; little or no regulatory oversight over the fields of traditional medicine and complementary treatments; and a lack of promotion by governments of the availability and efficacy of ARVs.

In some cases, government endorsement of unproven 'cures' has created confusion regarding the legitimacy of AIDS medicines, and governments and international organizations have done a poor job separating out their appropriate recognition of the important role played by traditional medicines in physical and psychosocial care, [1,2] from the dangerous and modern day hucksterism that AIDS 'cures' represent.

It is important at the outset to differentiate between legitimate and fraudulent promotion of herbal and other remedies. While there is very little data documenting the global use of herbal remedies, nutritional supplements, and traditional medicines, they are believed to be widely used by PLWHA to enhance their wellbeing. Unproven AIDS 'cures', on the other hand purport not just to enhance wellbeing but to specifically eradicate HIV. Less clearly definable is the promotion of HIV treatments which fall short of promising a 'cure' but advertise specific health benefits or biologic effects. Some of these, for example those that promote general immune boosting, may be considered akin to a supplement. Others, such as those which suggest an impact on HIV viral load are more properly considered a medicine or 'treatment'. Despite being promoted as 'traditional' or 'herbal', many supplements, 'cures', and treatments are produced using modern mass manufacturing techniques and with industrial chemicals.

Examples of unproven AIDS 'cures' and treatments can be found in the United States, Zambia, Mexico, South Africa, Thailand, India, Zimbabwe, and elsewhere. In the US for example, an orthopedic surgeon promoted an intravenous product containing aloe vera to treat AIDS and cancer, netting as much as US$18,000 for each 2-week treatment [3]. In Zambia, a 'cure' for AIDS called Tetrasil was promoted by a Zambian newspaper editor who held an ownership stake in the product with a prominent US AIDS denialist. The product was found to be a pesticide used to clean swimming pools [4]. Some PLWHA abandoned their ART for the drug [5]. 'Oxytherapy', using ozone, has been touted as a treatment for both cancer and AIDS in countries as diverse as Mexico and South Africa [6], and hyperthermia via high temperature baths has been increasingly promoted. In 2006 the South African drug regulatory agency said that "unregistered products claiming to cure HIV/AIDS, tuberculosis and various forms of cancer were readily obtained from street vendors" [7]. In Thailand, the Salang Bunnag Foundation handed out thousands of bottles of 'V-1 Immunitor', which was promoted as a cure for AIDS [8]. In Zimbabwe, the herbal remedy '*guandamiti*' – developed with support from the United Nations Educational, Scientific and Cultural Organization (UNESCO), Kellogg Foundation, the US National Cancer Institute and the World Health Organization – has been promoted as significantly reducing viral load (variously reported as 60–76% or 90%) and opportunistic infections [9]. None of the organizations supporting its development have spoken out against its unproven claims.

Despite widespread anecdotal evidence of openly marketed AIDS 'cures', little quantitative research has been done to assess the use of unproven treatments by PLWHA or the overlapping or concomitant use of ART and traditional medicines. One survey of PLWHA in India found that 64% had heard about a traditional treatment to cure HIV. Of those who had heard of a 'cure', 78% had tried the treatment [10]. By contrast, a study in Gabon of current patients at a medical clinic found that traditional healers were consulted first after the onset of HIV symptoms only 5% of the time, but 17% of patients sought concomitant therapy with traditional medicine and 3% sought faith healing [11]. Ethnographic research in Uganda [12] and South Africa [13] suggest a complex and chaotic process of treatment seeking on the part of PLWHA.

### Government responses to AIDS 'cures'

The most common response by governments to the problem of unproven AIDS treatments has been inaction. Due to the initial unavailability of ARV drugs, many governments did not promote ART as effective and life-saving and warn that other treatments are unproven and potentially dangerous. But even as ARV drugs have become more available, their still limited supply and the often complex and politically charged relationship between governments and traditional medical providers has caused governments to hesitate from aggressively promoting ART and challenging other claims. Even those governments that adopt pragmatic and 'progressive' AIDS policies promoting the open discussion of sexuality or the promotion of women's rights may find that criticizing unproven AIDS cures can be misunderstood as criticizing traditional medicines and can be politically risky. Consequently, most governments do nothing as 'cures' are promoted openly – in newspapers, on the radio, on billboards and other outlets.

In some cases governments have acted under pressure from civil society or the media. In Zambia, the government investigated the promotion of the purported 'cure' Tetrasil, and banned the advertisement and sale of the drug. In addition, a law was passed requiring that all drugs and natural remedies be tested for safety and efficacy, whether they are medicines or locally available herbs [5]. In Thailand, in response to the promotion of V-1 Immunitor, the Thai Ministry of Health tested 50 patients taking the medicine, and reported that the pills had no effect on their immune systems. Despite the fact that the pills were being promoted as a 'cure', the government found that the pills were not toxic and could be marketed as a food supplement [8].

In three cases, governments have directly promoted unproven treatments. The President of The Gambia has promoted a product which he claimed could cure AIDS, diabetes and asthma [14]. Iran's health minister, Kamran Baqeri Lankarani, announced that Iranian researchers had developed a medicine made completely with Iranian native plants that boosts the immune system and slows the replication of HIV [15]. Although the Minister does not appear to have referred to the remedy as a "cure", press reports from the region do [16] and it has been promoted by the Iranian Institute for AIDS Research as a "therapeutic vaccine" [17]. In both The Gambia and Iran, allegations have been made that government health officials have switched people to these unproven remedies from ART without their consent. Perhaps the most well known example of governments promoting alternatives to effective ARV medicines has been the government of South Africa, where President Thabo Mbeki and his health ministers have notoriously used their authority to challenge the use of ART and promote unproven therapies [18].

#### The Gambia

The claim by Yahya Jammeh, President of The Gambia, that he can cure HIV exemplifies the flagrant abuse of power and influence by a government leader. President Jammeh claims his secret concoction of herbs will cure AIDS in three days if people taking the treatment also discontinue taking ART and refrain from alcohol, caffeine and sex [19]. The consequences of criticizing the President have proven risky. Two journalists were fired by the owner of a pro-government newspaper in February 2007 after writing about the 'cure' [20]. Although reinstated, one of the journalists was subsequently fired by the paper in July after writing about it again [21]. A more prominent critic, the UN resident coordinator in The Gambia, Fadzai Gwaradzimba, was permanently expelled for asking for scientific proof of the treatment's effectiveness in a TV interview [22]. She also pointed out that his claims of a cure might encourage people to engage in risky behavior.

President Jammeh also turned to a respected Senegalese AIDS scientist, Prof. Souleyman Mboup, for support of his claim of a 'cure'. Jammeh publicly announced that tests conducted by Mboup confirmed that the treatment was successful. But in a statement released through the International AIDS Society (IAS) and the Society for AIDS in Africa, Mboup refuted this assertion and said that a visiting laboratory technician had independently run tests in his lab, and that no conclusion of effectiveness could be drawn [23].

In response to Jammeh's claims, the WHO and UNAIDS issued a statement reiterating that there is no cure for AIDS and warning that substituting untested remedies for evidence-based treatments could have adverse effects [24]. The statement encouraged President Jammeh to collaborate with international efforts to scientifically assess the safety, efficacy and quality of the remedy, but Jammeh refuses to allow samples of his herbs to be removed from the country to be tested. The WHO/UNAIDS statement appears to have been the extent of international pressure: the UN replaced the expelled envoy and has issued no further public statements.

#### Iran

On February 11, 2007, the President of Iran, Mahmoud Ahmadinejad, announced a series of technological and medical breakthroughs and asserted the country's nuclear "rights". Within this highly politicized announcement, President Ahmadinejad announced a new treatment for AIDS [25]. The treatment, an herbal medicine made from seven "completely native" Iranian herbs, was named IMOD, an abbreviation for "immuno-modulator drug", and developed by a Russian scientist and tested by the Iranian Research Center for HIV/AIDS. The Center's English language website does not directly state that the herbal remedy is a 'cure' but suggests that IMOD could be used as a "therapeutic vaccine" and as the "first choice" for treatment in resource constrained developing countries [17]. At the announcement ceremony, Iran's top AIDS expert and head of the research center, Minoo Mohraz, stated that, "a medicine that is used for *curing AIDS *[emphasis added], should be completely secure and reliable", and stated that this is true in the new medicine [15].

The announcement of the new treatment by President Ahmadinejad was accompanied by the awarding to the Minister of Health a national medal of honor, while scientists protesting the testing of the remedy against a placebo control group have been censured or fired. The President's Office for Technology Cooperation has subsequently promoted the remedy and sought partners for joint marketing, clinical trials and manufacturing, and stated that it was available for export.

Four months after the first public announcement of the remedy, Christian Salazar, UNICEF's coordinator on HIV in Iran, praised the country's "progressive and pragmatic" efforts in fighting the virus that causes AIDS, but said nothing about IMOD [26] and no further comments from UNAIDS, WHO or other governments have been made.

#### South Africa

The government of South Africa's unwillingness to acknowledge the effectiveness of ART and outright questioning of the relationship between HIV and AIDS has been extensively reported [18]. The consequences to public health in terms of the delayed introduction of effective medicine to treat HIV infection and the continuing lack of trust in ART has been less well documented, as have the direct relationship between South African government officials and promoters of vitamins and herbal remedies. Three noteworthy examples include the cases of 'Virodene', an industrial solvent promoted by a pair of University of Pretoria scientists, and the nutritional supplements and extracts promoted by the German Matthias Rath and the Dutch Tine van der Maas.

In early 1997, the Minister of Health, Nkosazana Dlamini-Zuma, met with two University of Pretoria scientists promoting 'Virodene' a drug composed of the toxic industrial solvent dimethylformamide as a 'cure' for AIDS [27]. The Health Minister in turn invited the scientists to a cabinet meeting where cabinet ministers heard from the scientists and patients who had taken the drug as part of an unauthorized clinical trial. Cabinet Secretary Jakes Gerwel was impressed by the effectiveness of the 'cure': "*The patients said they were dying, they got this treatment, and then they were saved!" *[28].

Thabo Mbeki, acting as the deputy President, chaired the meeting, and shared the enthusiasm of Gerwel. However, a major obstacle to the further promotion of the 'cure' was the refusal by the government's independent Medicines Control Council (MCC) to provide permission for the continuation of the clinical trial, due to the known toxicity and lack of effectiveness of dimethylformamide. Eventually the Minister of Health was able to replace key MCC members [27], however official approval was still not provided. Nonetheless, the researchers continued, without ethical approval, to test the drug in South Africa [6] and Tanzania [29].

Both Mbeki and his two health ministers have referred to ART as 'toxic' and promoted alternatives to ART, including garlic, olive oil, beetroot and lemon. The doctor Matthias Rath who has offices in Germany, the Netherlands and California, has had close ties to Mbeki and his administration. In 2005 Rath promoted his nutritional supplements and criticisms of ART via newspaper advertisements in South Africa (entitled "Stop AIDS Genocide by the Drug Cartel") and directly in poor townships around Cape Town. Despite the resulting controversy and calls for a government response, the Ministry of Health refused to contest the claims and unequivocally defend the effectiveness of ART [30].

The Minister of Health also promoted an herbal treatment developed by a Dutch nurse composed of African potato extract, olive green leaf extract, and grapefruit seed extract called 'Africa's Solution'. Tshabalala-Msimang invited the remedy's developer to address provincial health ministers and arranged for the provision of the solution to AIDS patients in government hospitals and clinics[31]. Allegedly, the solution was given to over 40,000 people, but the records were destroyed when a burglar urinated on them [32]. Tshabala-Msimang also arranged for a Health ministry advisor to work directly with the manufacturer at government expense [33]. At the 2006 International AIDS Conference in Toronto, the official South African government display booth included garlic, lemon and beetroot. Only at the protest of South African AIDS activists were boxes of ART added.

There has been considerable speculation as to the motivation of Mbeki and Tshabalala-Msimang. Some have questioned whether the rejection of the use of AZT might be due to a desire to promote nationally-developed drugs [34]. It was also reported that Mbeki's party, the African National Congress, was to receive 6% of the shares in CPT, the company that manufactures Virodene [35]. South African newspapers have reported that tens of millions of South African Rand were paid for the Virodene trials directly from the Office of the Presidency [36].

The UN's response in South Africa has been typical: issuing occasional press releases but not otherwise seeking to counteract the promotion of unproven AIDS 'cures'. UN agencies in South Africa called Rath's advertisements "wrong and misleading" and said Rath's implication that the UN agencies supported his nutritional approach were "dangerous and unhelpful" [37] but have been silent on other proposed 'cures' such as Ubejane or Secomet V, both widely promoted as 'cures' in the past two years. Meanwhile, HIV test counselors and health clinic staff participate in the promotion of unproven 'cures' and supplements [38].

### Counterfeit ARVs

According to the WHO, a counterfeit drug is "one which is deliberately and fraudulently mislabeled with respect to identity and/or source. Counterfeiting can apply to both branded and generic products and counterfeit products may include products with the correct ingredients or with the wrong ingredients, without active ingredients, with insufficient active ingredients or with fake packaging" [39]. Counterfeited drugs may have little or no therapeutic value, causing illness and death from the condition supposedly being treated, or the fake drugs may be composed of toxic substances that directly cause illness and death. Counterfeited drugs with the appropriate active ingredients in subclinical amounts can also lead to prolonged illness or death, but pose the further risk of encouraging the spread of drug resistant pathogens. Although much counterfeit drug trade occurs in the unregulated market of unofficial drug vendors, especially in developing countries, counterfeit drugs are also found extensively in licensed pharmacies.

Counterfeit operations range from small cottage industries to large factories and can involve smuggling and illegal importation, use of fictitious businesses and front companies, and false documents. Counterfeiters have become extremely sophisticated in mimicking branded medicines, creating nearly identical packaging and pills and using false batch code information. These fake drugs easily pass customs inspections and are accepted and distributed by pharmaceutical supply companies and pharmacies.

Like unproven 'cures', the true extent of counterfeit drugs has not been fully documented. Investigative journalism provides the preponderance of information on counterfeiting compared with relatively scant treatment in the scientific and public health literature [40]. However, unlike unproven 'cures', governments and international agencies have estimated the prevalence of counterfeit medicines. The US Food and Drug Administration estimates that counterfeit drugs account for 10% of the international market and according to the WHO the proportion may be 25% of the market in developing countries [41]. The International Narcotics Control Board has warned that up to half of all prescription narcotics in developing countries may be counterfeit [42]. In Nigeria, before 2006, health officials estimated that 70% of drugs in circulation in the country were either fake or adulterated [43].

It is difficult to assess the true prevalence of counterfeit ARVs, in part because of the lack of specific research, but also because anecdotal reports of counterfeit ARVs are sometimes mixed with reports of unproven cures or authentic ART smuggled from other countries or illegally diverted from medical supplies. Nonetheless, a number of factors make ART an attractive target for counterfeiters, especially: high unit costs and long-term, sustained demand. In addition, stigma and fear of loss of confidentiality in health care settings increase demand for ARVs delivered through often poorly regulated private sector health care providers, pharmacies or other channels.

In 2004 the Kenyan newspaper 'The Nation' documented an active informal market for ARVs, including AIDS cures and counterfeit drugs, in Tsavo Road in Nairobi [44]. A 2003 report from Ethiopia found illegally imported "concoctions" being marketed as ART [45]. With the collapse of the economy and medical system in Zimbabwe have come reports of ART stolen from the public health care system and smuggled from neighboring countries [46] and individuals switching from ART to herbal remedies[47]. The Zimbabwean Health and Child Welfare Minister said that ART were being sold at flea markets and hair salons and called on civil society organizations to help "evaluate" the problem. In Burma, where it is estimated that only 7% of individuals living with HIV who need ARVs have access to them [48], the UNAIDS Country Coordinator stated that he was aware of no programs to monitor for counterfeit ARV drugs. Assessing the risk of the emergence of counterfeit ARVs in the country, he stated: *"Everything else in Burma is counterfeit. It's only a matter of time before ARVs are too." *[B. Williams, personal communication]

The trafficking of counterfeit ARVs has been specifically documented in two cases to date. In 2003 the WHO issued an alert that a product called 'Ginovir 3D', marketed in Cote d'Ivoire as a triple ARV combination product, contained only one of the drugs (zidovudine) on the label, none of the other 2 drugs (lamivudine and indinavir), and an additional drug not on the label (stavudine) [49]. In 2004 Medecins Sans Frontieres (MSF) discovered counterfeit ARVs on the market in the Democratic Republic of Congo that contained an antidepressant and a muscle relaxant [50]. Whether the lack of more recent cases is due to the increased availability of ART or some other factor, or is simply due to the lack of close monitoring is unknown.

Unlike the merely rhetorical response by governments and UN agencies to unproven 'cures', a range of actions have been taken at both levels to combat the proliferation of counterfeit medicines. Governments have adopted and refined legislation opposing counterfeiting and have targeted specific highly counterfeited medicines with special protections (including packaging, price management, regulation, law enforcement). International agencies have led regional and international initiatives aimed at sharing information and reporting incidences of trafficking, and have provided technical support to countries and regional coalitions.

To address the high levels of the counterfeit anti-malaria drug artesunate in Thailand, most malaria is treated at no cost in government malaria clinics where drug supplies can be more closely monitored [51]. In China, national pharmaceutical laws were strengthened in 2001, including strict controls on price management, manufacturing registration, import inspections, and law enforcement [52]. However, corruption and the lack of protection for whistleblowers undermined China's attempt to establish a more rigorous drug regulatory system, and the 2001 laws did not apply to drugs manufactured in China for export [53]. A series of scandals involving counterfeit pharmaceutical exports in 2007 led to intense international pressure on the Chinese government resulting in the conviction and subsequent sentencing to death of the country's two top drug regulators for accepting bribes [54,55].

When governments have been more aggressive towards counterfeiting syndicates, the result has often been violence. Enforcement officers in India [56] have been threatened and shot at, and Dora Akunyili, the head of the Nigerian National Agency for Food and Drug Administration and Control (NAFDAC) faced threats and violence when she initiated an aggressive crackdown on counterfeit medicine in 2000 [57]. In 2001 the NAFDAC laboratory was vandalized and a policeman on duty killed. In 2003 an assassination attempt was made on Akunyili's life.

WHO has proactively tried to establish international norms regulating pharmaceuticals and addressing the trafficking of counterfeit products, although the result has been less than fully successful. In the 1980s, the WHO initiated a voluntary, confidential international system to report counterfeiting. However, countries were reluctant to report and only a few did so [40]. In 1999, the WHO developed guidelines to assist countries in implementing effective regulation and monitoring of counterfeit medicines [58]. In addition, a series of regional efforts were launched to increase national capacity to combat counterfeiting, since most countries do not have the capacity to conduct the detailed chemical analysis required to determine the biological activity of imported drugs. For example, a 2003 WHO initiative funded by Australia in Cambodia, China, Laos, Burma, Thailand, and Vietnam aimed to combat counterfeiting in the region, to promote advocacy activities, and to strengthen inspection and postmarketing surveillance [59]. In 2006 the WHO created the International Medical Products Anti-Counterfeiting Taskforce (IMPACT) to increase international coordination of efforts to monitor and combat counterfeit medicines. However, like earlier, failed initiatives, participation is voluntary and nonbinding [60].

## Discussion

Almost 2 billion people lack access to essential medicines [61]. Improving access to existing medicines could save 10 million lives each year [62]. Scientists, AIDS activists and governments around the world responded with outrage to the South African government's refusal to acknowledge that HIV caused AIDS and that ARVs were effective and life-prolonging. Yet that outrage has been inconsistent, and the outcry in response to counterfeit medicines and the promotion by the Presidents of The Gambia and Iran of unproven AIDS remedies has been muted.

### Government and UN Obligations

International legal standards impose on governments a responsibility to respect, protect and fulfill certain enumerated rights of individuals. The right to life, to be protected from harm and exploitation, the right to the progressive realization of access to health services, and the right to receive and impart information are all relevant to protection from unproven AIDS treatments and counterfeit medicine.

The international recognition of the right to health began with a reference to health in the United Nations Charter and was affirmed in the preamble to the 1946 Constitution of the World Health Organization. The WHO Constitution, in Article 21, empowers the World Health Assembly to adopt regulations that are binding on Member States related to, among other things, the safety, purity and potency and the advertising and labeling of biological, pharmaceutical, and "similar products" moving in international commerce [63]. The global trafficking of counterfeit medicines clearly falls within this protection, and, as we have seen, unproven AIDS treatments are commonly promoted, tested, and shipped internationally as well. The promotion of unproven 'cures' though, even when not involving international commerce, can also be covered by Article 19 of the WHO Constitution, which states that the World Health Assembly "shall have the authority to adopt conventions and agreements with respect to any matter within the competence of the Organization". Article 19 was the basis for an international framework convention on tobacco control, which was first proposed in 1996, was adopted by consensus by WHO's 192 member states in 2003, and entered into force in February 2005 [64].

UN guidelines on HIV/AIDS and human rights emphasize the need for states to take affirmative action to provide adequate, accessible and effective HIV-related prevention and care education, information and services. The guidelines say specifically:

*States should take legislative and other measures to ensure that medicines are supplied in adequate quantities and in a timely fashion, and with accurate, current and accessible information regarding their use. For example, consumer protection laws or other relevant legislation should be enacted or strengthened to prevent fraudulent claims regarding the safety and efficacy of drugs, vaccines and medical devices, including those relating to HIV *[65].

In 2006, the United Nations issued a declaration on HIV/AIDS committing all member states to pursue "all necessary efforts" to scale up HIV/AIDS treatment programs towards the goal of providing universal access to HIV/AIDS treatment by 2010 [66]. UN member states have also committed via the Millennium Development Goals to work in cooperation with pharmaceutical companies to ensure access to affordable essential drugs in developing countries [67]. Reflecting on these commitments and international human rights law, the UN Special Rapporteur on the Right to Health, Paul Hunt, has said unequivocally that: "access to medicines forms an indispensable part of the right to the highest attainable standard of health" and that the absence in states of regulation of medicines is "clearly inconsistent with the right to the highest attainable standard of health" [68].

### Steps forward

The first step towards addressing unproven AIDS treatments and counterfeit drugs must be to increase our knowledge and understanding of the problem. The distribution of medicines and 'cures' in developing countries has long been recognized as chaotic and occurring through commercial and other informal networks in addition to health care settings [69-73]. A number of researchers have also made the observation that most pharmaceuticals, even ones intended to be strictly controlled, are largely self-regulated [74,75]. More research must therefore be conducted on the prevalence of unproven 'cures', treatments and supplements, and on PLWHA treatment-seeking behavior, specifically on the extent to which unproven remedies delay uptake of ART or complement it. Governments must conduct periodic monitoring of the availability of unproven treatments on the market and the claims made by promoters of such treatments. The authenticity of ARV drugs must be regularly monitored.

The second step is to continue to aggressively scale-up ART programs and to widely promote their availability – to all who need it, in an equitable and non-discriminatory manner. Through the widespread promotion of ART and expanding routine HIV testing linked to treatment, Botswana has been able to achieve nearly universal knowledge of, *and confidence in *ART as an effective treatment for HIV/AIDS [76]; achieving the delivery of ART to 85% of those in need [77]. In Zambia, by contrast, less than 30% of women and men who need ART receive it [78], and unproven AIDS 'cures' are common [4]. Most countries will continue to fall short of the level of access to ART provided in Botswana, and uneven access will facilitate the promotion of unproven medicines.

In most countries, information on ART is provided passively, by health care providers to those who are able to access treatment. Outreach, provided through treatment literacy campaigns emphasizing the effectiveness of ARV drugs, the importance of adherence to prescribed regimens, and the danger of untested 'cures' have been insufficiently funded, and have been largely implemented by NGOs and networks of PLWHA. To be effective, these campaigns must be expanded and conducted through multiple channels, involving NGOs and PLWHA networks, but also public and private sector Western medical providers, traditional healers, and mass media.

A third step would be for governments to enact or revise legislation against counterfeit medicines and the fraudulent promotion of unproven remedies. States must cooperate with international efforts to combat the trafficking of counterfeit medicines and unproven cures. Alternatively, the issue of counterfeit medicines and unproven remedies could be addressed within a framework convention on global health. Advocates for such a convention [79] stress that this approach can foster capacity building of developing country health systems and facilitate international cooperation towards addressing infectious disease control and meeting basic health needs. Explicitly incorporating counterfeit drugs and unproven therapies into the convention could provide the fight against such treatments with a universal definition of terms, the wider dissemination of reports of counterfeit drugs and unproven therapies, and a requirement on states to report to the international body steps taken to restrict the dissemination of such drugs and therapies.

Finally, WHO and UNAIDS must more forcefully promote the message that only ARV drugs have been proven to be effective against AIDS, and must regularly and consistently speak out and challenge governments that promote unproven 'cures' or fail to regulate claims of AIDS treatments. Following the publication of a letter in November 2007 by Human Rights Watch to the Kamran Baqeri Lankarani, Iran's Minister of Health and Medical Education concerning the promotion of IMOD [80], the website promoting the 'cure' was taken down.

### AIDS treatment in context

While the lack of universal access to ART is one factor in the proliferation of unproven AIDS 'cures' and counterfeit drugs, there are much broader considerations. ART, even when recognized as life-saving and when widely available, can not 'heal' all wounds experienced by those infected by HIV. The promoters of unproven AIDS remedies – be they traditional medical providers, faith healers, or Presidents – often prey not only on the lack of understanding of ART, but the broader fears and hopes of PLWHA for respect, dignity, and a healthy future free from dependence, stigma and discrimination. ART programs inevitably exist in the political sphere and, as we have seen, governments use the promotion of unproven AIDS remedies as part of larger political campaigns to express benevolence or promote 'indigenous' solutions.

Paul Farmer has written eloquently about the introduction of ART in a rural clinic in Haiti, emphasizing the hope felt by his patients upon receiving the drug and experiencing the 'Lazarus'-like effects of it [81]. But Farmer has also spoken about how the clinic's efforts include broader social support and how this act of providing care to an impoverished population can create a radical shift in perceived stigma.

Contrast Farmer's experience with that of modern, global, faith healers such as the Canadian Benny Hinn who promises deliverance from a wide range of ailments as part of a ministry that is alleged to have revenues of $100 million per year. In 2007 Hinn preached in Uganda to a crowd of 50,000 people at a football stadium. A journalist attending the event interviewed a 19 year old woman living with HIV who attended. She described how she was living with her brother after her parents kicked her out of her home when they discovered she was infected with HIV. The article did not identify if the woman was taking ART, but she clearly longed for more than just the medical management of her condition. She said of Hinn: *"He cured me of AIDS, I can feel it. I just know"*[82].

Everyone, everywhere, involved in the fight against AIDS wants there to be a cure, and a cure as simple and as painless as words spoken to a crowd at a football stadium. But until a cure is found, ART represents our only form of life-saving treatment. States and the global community have committed billions of dollars to increasing access to effective medicine to fight AIDS, but have done little to address unproven AIDS drugs and the threat of counterfeit ART. The extent to which this failure represents actual harm is as yet largely unknown, but the existence of it is an unfortunate testament to the limitations of governments to fulfill their mandates to protect from harm and realize the right to health of those living with HIV/AIDS.

## Competing interests

The author(s) declare that they have no competing interests.

## Authors' contributions

JA conceived and conducted the research and wrote the manuscript.
